# A Unique Case of Testicular Plasmablastic Lymphoma in a Patient with Human Immunodeficiency Virus

**DOI:** 10.7759/cureus.4839

**Published:** 2019-06-05

**Authors:** Carmel Moazez, Surabhi Amar

**Affiliations:** 1 Oncology, Creighton University School of Medicine/Maricopa Medical Center, Phoenix, USA; 2 Oncology, Maricopa Medical Center, Phoenix, USA

**Keywords:** testicular, plasmablastic lymphoma, hiv, plasmacytic lymphoma

## Abstract

Plasmablastic lymphoma (PBL) is a rare, aggressive, diffuse large B-cell lymphoma usually arising in the oral cavity of human immunodeficiency virus (HIV) patients. Here we describe a patient with HIV who presented with cutaneous nodules that were biopsied and found to be positive for PBL, but whose primary source was found to be testicular. This is the first reported case of a patient with HIV who presented with cutaneous nodules that tested positive for PBL, but whose primary source of neoplasm was found to be testicular.

## Introduction

Plasmablastic lymphoma (PBL) is a rare, aggressive B-cell lymphoma first described by Delecluse et al. in 1997 [1]. In human immunodeficiency virus (HIV) patients, 2.6% of non-Hodgkin's lymphomas are found to be due to PBL. HIV patients have a 60 times greater risk of acquiring a non-Hodgkin’s lymphoma compared to the general population. PBL is mostly reported in the oral cavity of HIV positive patients especially those with poor disease control. It has also been reported in the lungs, nasal cavity, gastrointestinal tract, lymph nodes, and rarely as cutaneous nodules. Extra-oral PBL is rare and only a handful of cases arising in the testis have been described. The pathogenesis is thought to be due to prolonged B-cell stimulation and proliferation [2]. This tumor is rapidly progressive and often involves the central nervous system (CNS) either at initial presentation or at relapse, as in our patient [3]. Here we describe a case of a patient with poorly controlled HIV who presented with new onset cutaneous nodules and was found to have primary testicular PBL.

## Case presentation

**Figure 1:**
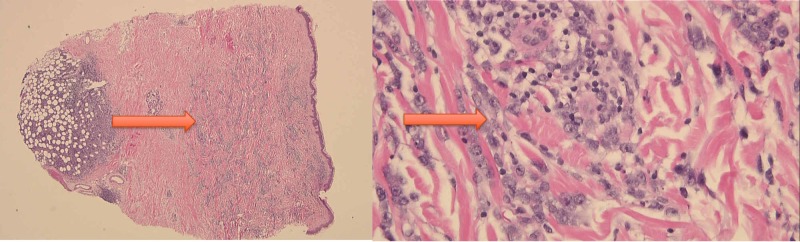
Biopsy result of the largest skin nodule on the abdomen showing plasmacytic/plasmablastic neoplasm and “starry sky” appearance

A 55-year-old male with poorly controlled HIV infection (cluster of differentiation (CD)4 count 194) and severe pulmonary hypertension (HTN) due to amphetamine abuse presented with a two-week history of cutaneous nodules (eight to ten nodules on the abdomen with the largest being 2 x 2 cm). He did not have any peripheral lymphadenopathy or hepatosplenomegaly. A biopsy of one of the nodules revealed plasmacytic/plasmablastic lymphoma which was CD38, CD45, Epstein-Barr virus positive, and CD20, CD138 negative (Figure 1).

**Figure 2:**
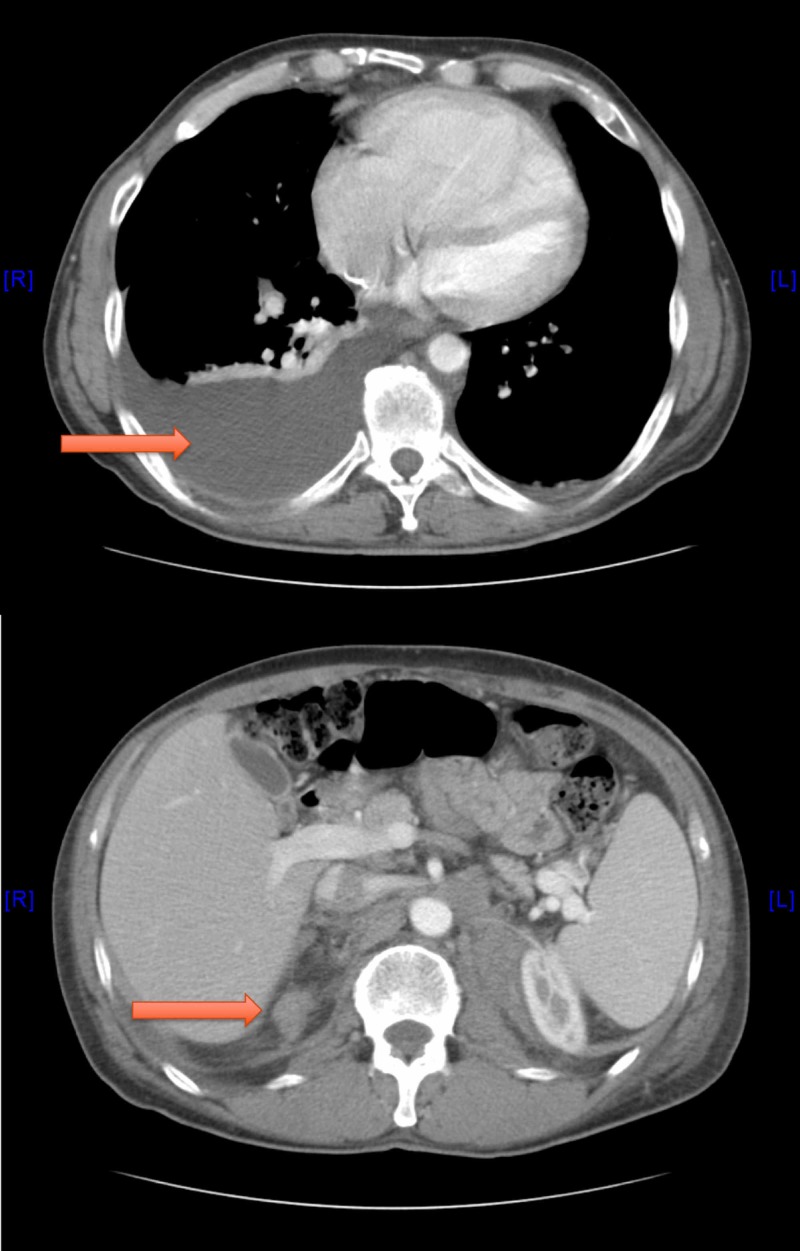
Computed tomography (CT) of the chest/abdomen/pelvis (axial view) showing right pleural effusion and left retroperitoneal mass

Computed tomography (CT) of the chest/abdomen/pelvis showed a large right pleural effusion, small left pleural effusion, a 5-cm left retroperitoneal mass, smaller right retroperitoneal mass, and mesenteric lymph nodes (Figure 2).

**Figure 3:**
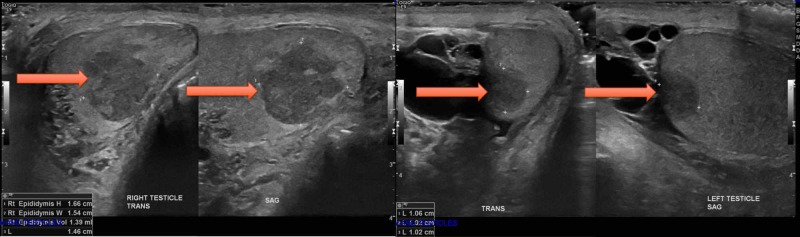
Ultrasound images of bilateral testicles The right testicle has a 1.6 x 1.5 x 1.4 cm vascular mass and 2.8 x 2.2 x 1.9 cm epididymal cyst. The left testicle has a 1.1 x 1.0 x 1.0 cm vascular hypoechoic mass and 4.3 x 3.4 x 2.6 cm epididmal cyst.

He had a right-sided thoracentesis and cytology was consistent with plasmacytic/plasmablastic cells. The scans also suggested bilateral testicular enlargement prompting a testicular ultrasound which showed bilateral testicular masses (Figure 3).

At this point, the primary source of malignancy was thought to be testicular. Unfortunately, due to the patient’s severe pulmonary HTN, he was unable to undergo anesthesia for a testicular biopsy as he kept becoming hypoxic. Since this lymphoma has a high risk of relapse in the CNS, a spinal tap was done but the fluid was negative for malignant cells. He was treated with cyclophosphamide, vincristine, and prednisone (CVP) regimen along with high dose methotrexate (to provide CNS prophylaxis). He had a complete response after five cycles of chemotherapy.

Eight months after his initial diagnosis, he presented to the clinic confused and forgetful. Further work up revealed the involvement of cerebral spinal fluid with lymphoma. He underwent an Ommaya placement with intrathecal chemotherapy. His spinal fluid became negative for malignant cells after three treatments. Bilateral testicular radiation therapy was given (as testes have poor chemotherapy penetration and can act as sanctuary sites). In spite of all this, he developed delirium and acute hypoxic respiratory failure and continued to decline and died ten months after his diagnosis.

## Discussion

Here we describe a patient with HIV who presented with cutaneous nodules that were biopsied and found to be positive for PBL. After imaging, his primary source was found to be testicular. Badyal et al. described a case of testicular PBL in an HIV patient who presented with bilateral scrotal swelling and no cutaneous nodules [4]. Our case is unique as it is the first case of primary testicular PBL that presented with cutaneous nodules.

Risk factors for non-Hodgkin's lymphoma in HIV patients include male gender, low CD4 count, high viral load, and increased age [4]. PBL has features of B-cell lymphoma and plasma cell neoplasms. Therefore, diagnosis and treatment of this aggressive disease has been difficult. PBL has been associated with Epstein-Barr virus infection. Biopsy should show a “starry sky” pattern with frequent macrophages. Immunostaining is usually positive for CD38, CD138, slightly positive for CD45, negative for CD19 and CD20 although variations can occur [3]. Our patient was positive for CD38, CD45 and negative for CD138 and CD20. 

Currently, there is no standard of care for treatment. Current guidelines are recommending more aggressive therapy than just cyclophosphamide, doxorubicin, vincristine, prednisone (CHOP) in these patients. Infusional etoposide, vincristine, doxorubicin, cyclophosphamide, prednisone (EPOCH) and cyclophosphamide, vincristine, doxorubicin, high-dose methotrexate/ifosfamide, etoposide, and high-dose cytarabine (CODOX-M-IVAC) are considered better treatment options [4-5]. Intrathecal prophylaxis is also recommended particularly in patients with HIV due to the high rate of proliferation in PBL [6].

The prognosis of PBL is poor. Although common in HIV patients, PBL may also occur in a non-HIV group. Tchernonong et al. found that HIV patients had a better prognosis than non-HIV patients. This was thought to be due to immune surveillance restoration by combination antiretroviral therapy (cART) and their younger age which allowed them to receive more aggressive chemotherapy [7]. Median survival in HIV patients was found to be 10 to 15 months and three-year survival was found to be 25%. Patients with HIV who did not receive treatment had a median survival of three months [4]. Antiretroviral therapy is recommended in patients with PBL to prevent further disease progression and has been shown to improve rates of remission when used in combination with chemotherapy [5-6].

## Conclusions

PBL is a rare tumor that is most commonly found in patients with HIV. It usually presents as an oral neoplasm, but extra-oral manifestations have been found. It rarely presents as cutaneous nodules or testicular neoplasm. Here we describe the first case of testicular PBL in an HIV patient manifesting as cutaneous nodules.
